# Risk factors for regional lymph node metastasis in rectal neuroendocrine tumors: a population-based study

**DOI:** 10.3389/fmed.2024.1383047

**Published:** 2024-09-04

**Authors:** Ruizhen Li, Xiaofen Li, Yan Wang, Chen Chang, Wanrui Lv, Xiaoying Li, Dan Cao

**Affiliations:** ^1^Department of Abdominal Oncology, Cancer Center, West China Hospital, Sichuan University, Chengdu, Sichuan, China; ^2^Abdominal Oncology Ward, Division of Medical Oncology, Cancer Center, State Key Laboratory of Biotherapy, West China Hospital, Sichuan University, Chengdu, Sichuan, China

**Keywords:** regional lymph node metastasis, rectal neuroendocrine tumors, SEER, West China hospital databases, tumor size

## Abstract

**Introduction:**

The identification of risk factors for regional lymph node (r-LN) metastasis in rectal neuroendocrine tumors (R-NETs) remains challenging. Our objective was to investigate the risk factors associated with patients diagnosed with R-NETs exhibiting r-LN metastasis.

**Methods:**

Patient information was obtained from the Surveillance, Epidemiology, and End Results (SEER) database, complemented by data from the West China Hospital (WCH) databases. The construction cohort comprised patients diagnosed with R-NETs from the SEER database, while cases from the WCH database were utilized as the validation cohort. A novel nomogram was developed to predict the probability of r-LN metastasis, employing a logistic regression model.

**Results:**

Univariate analysis identified four independent risk factors associated with poor r-LN metastasis: age (HR = 1.027, *p* < 0.05), grade (HR = 0.010, *p* < 0.05), T stage (HR = 0.010, *p* < 0.05), and tumor size (HR = 0.005, p < 0.05). These factors were selected as predictors for nomogram construction.

**Discussion:**

The novel nomogram serves as a reliable tool for predicting the risk of r-LN metastasis, providing clinicians with valuable assistance in identifying high-risk patients and tailoring individualized treatments.

## Introduction

Neuroendocrine tumors (NETs), which account for just 0.5% of all neoplasms (1, 2), typically develop in various organs, including the gastro-entero-pancreatic tract, lungs, gallbladder, thymus, thyroid, testicles, ovaries, and skin (3–5). Among the Asian population, R-NETs have the highest incidence rate of all gastroenteropancreatic neuroendocrine tumors (6, 7). Previous studies have established an association between r-LN metastasis in R-NETs and poorer prognosis (8). In addition to tumor size, the presence or absence of r-LN metastasis significantly influences treatment options. The European Neuroendocrine Tumor Society consensus guidelines recommend endoscopic resection for R-NETs ≤10 mm, provided there is no invasion of the intrinsic muscle layer or r-LN involvement (9). However, approximately 10% of patients present with r-LN metastasis, which necessitates radical resection (10–12). Diagnosis is typically made through computed tomography (CT) or endoscopic ultrasound (EUS). The Italian Society of Gastroenterology recommends that local staging for tumors larger than 10 mm, or in cases where complete resection is not feasible, should be evaluated using EUS (13). While 68Ga-DOTANOC PET-CT demonstrates satisfactory sensitivity in early r-LN metastasis detection, its widespread clinical application is hindered by high examination costs (14). Thus, identifying high-risk factors for r-LN metastasis is crucial for surgical planning. Given the existing controversies surrounding the prognostic significance and operative procedures for R-NETs with r-LN metastasis, our study conducts a comprehensive evaluation. We aim to determine preoperative factors predicting r-LN metastasis and analyze its impact on R-NETs, utilizing data from the SEER database.

Our study presents a detailed nomogram, developed based on the SEER database, to predict both r-LN metastasis and distant metastasis. This nomogram has been validated using a cohort from the WCH database.

## Patients and methods

### Patient characteristics and study design

In the construction cohort, the data of patients were extracted from the SEER database, which collected information on cancer. SEER cohort Data from patients with pathologically confirmed R-NETs were retrieved from the SEER database (from 1988 to 2014) using SEER*Stat version 8.3.4 software. In the validation cohort, cases were retrospectively obtained from the WCH database. The collected data included patient demographics, histology, T, N and M stage of the primary tumor, tumor size, r-LN metastasis and distant metastasis. Patients were retrieved according to International Classification of Diseases for Oncology (third revision) codes: carcinoid tumor (8240), argentaffin carcinoid tumor (8241), enterochromaffin cell tumor (8242), mucocarcinoid tumor (8243), mixed adenoneuroendocrine carcinoma (8244), adenocarcinoid tumor (8245), neuroendocrine carcinoid (8246), and atypical carcinoid tumor (8249). We included patients with primary tumors of the rectum. The exclusion criteria were as follows: patients with unknown TNM stage, unknown tumor size, unknown metastatic r-LN, unknown distant metastasis or unknown tumor grade by pathologic examination. We analyzed all patient data for demographic characteristics, including sex and age. Tumor-specific variables, including grade, size, metastatic r-LN, and distant metastasis, were evaluated.

### Statistical analysis

Student’s *t* test and the Chi-squared test (or Fisher’s exact test) were performed in univariate analysis depending on the categorical and ordinal variables. Multivariate analysis was performed using logistic regression analysis to evaluate potential risk factors, including age, sex, tumor size, TNM stage and histologic grade. Hazard ratios (HR) and 95% confidence intervals (CIs) were calculated. Statistical analysis was accomplished using SPSS 26.0 software (IBM, Armonk, NY, United States). For all analyses, *p* values <0.05 were defined as statistically significant.

A nomogram based on the results of multivariate analysis was constructed using the rms package in R version 4.1.3,^1^ which estimated r-LN metastasis and distant metastasis. The concordance index (C-index) was used to assess the predictive performance of this nomogram. Calibration curves were utilized to evaluate the agreement between the predicted and actual r-LN metastasis rates.

## Results

### Patient characteristics

**Figure 1:**
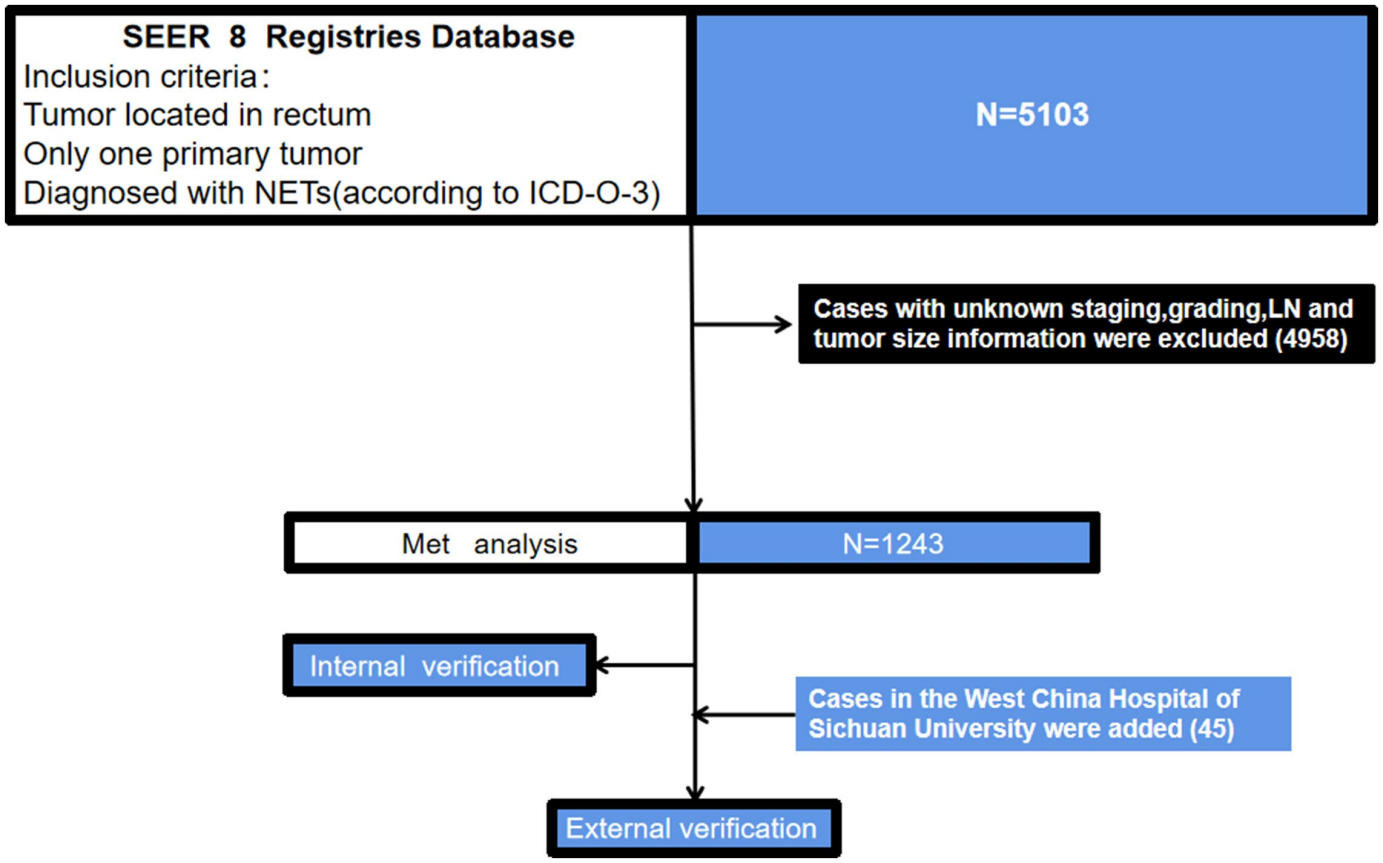
A flowchart of patient selection and study design. NETs, neuroendocrine tumors; ICD-O-3, international classification of diseases for oncology, 3rd edition; SEER, the surveillance, epidemiology, and end results dataset.

**Figure 2:**
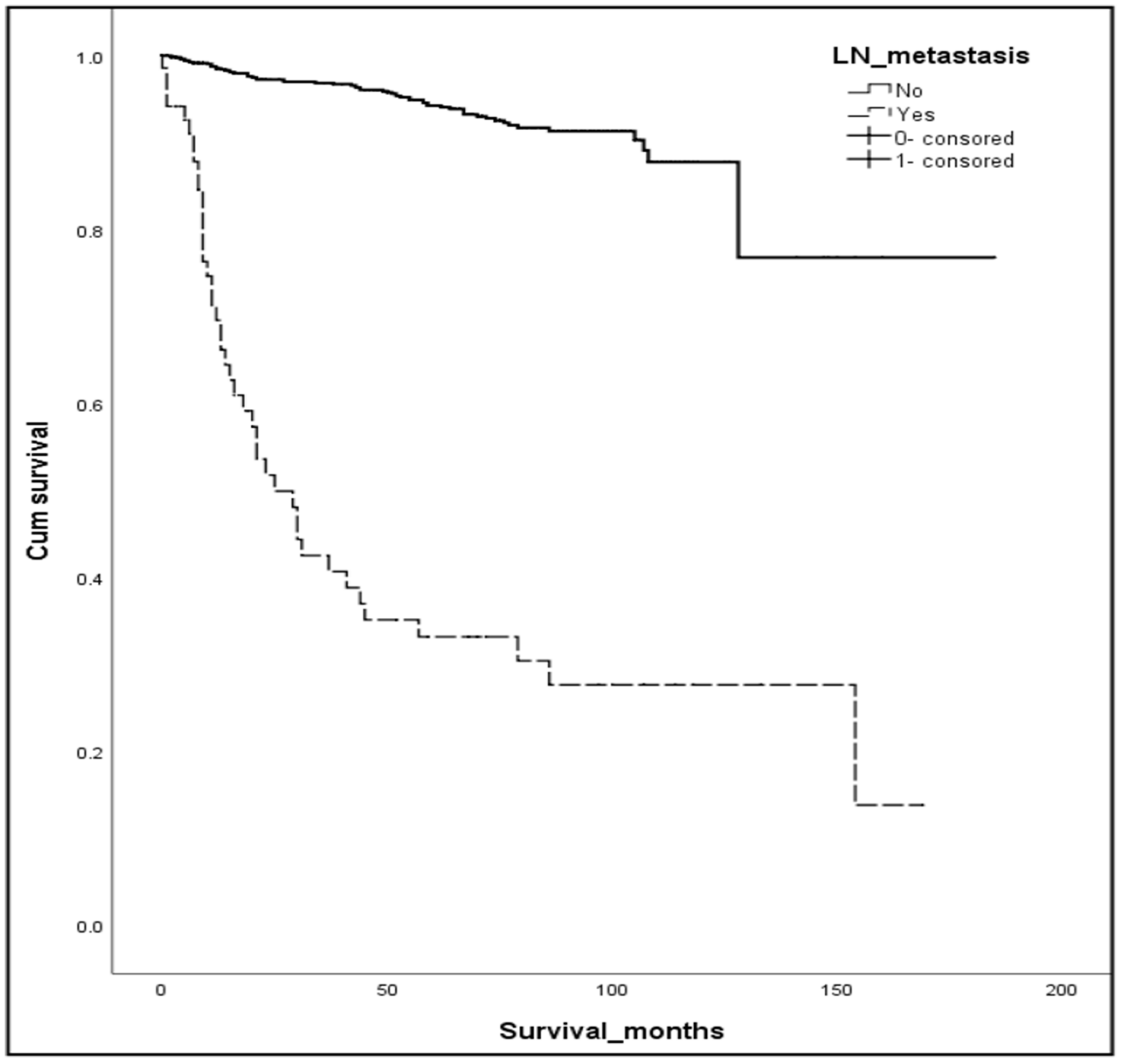
Kaplan–Meier curves of OS for patients with or without r-LN metastasis using the SEER database. Compared to negative r-LN metastasis, positive r-LN metastasis was significantly associated with worse OS (mOS: 21 vs. 58 months, 95%CI = 15.06–34.94, *p* < 0.001).

Using the previously mentioned criteria, we screened a total of 5,103 patients with R-NETs, excluding some due to missing data on essential staging, r-LN metastasis, and other factors. Ultimately, 1,243 newly diagnosed R-NET patients from the SEER database were included in the final analysis for the construction cohort (Figure 1). Similarly, 45 patients in the WCH database were assigned to the validation cohort (Figure 1). Clinicopathologic characteristics in the two cohorts regarding age, sex, tumor size, T stage, histologic grade, r-LN metastasis and distant metastasis. The mean age at diagnosis of R-NETs was 53 years old in the SEER cohort and 48 years old in the validation cohort. In the SEER database, more than half of the patients (53.4%) were males. There were 251 (20.2%) cases with tumor sizes greater than 1 cm, 1,115 (89.8%) at the T1 stage, and 1,034 (89.8%) at Grade I. r-LN and distant metastasis have only been shown in a few patients (5.7 and 3.4%, respectively). For the validation cohort, the rate of tumor size greater than 1 cm was 51.1%, and the r-LN and distant metastasis rates were 22.2 and 13.3%, respectively. The details are shown in Table 1. We analyzed the survival curves of patients with or without r-LN metastasis in the SEER cohort. The results revealed that median OS was considerably lower in r-LN metastasis patients than in patients without r-LN metastasis (21 vs. 58 months, 95% CI = 15.06–34.94, *p* < 0.001) (Figure 2).

**Table 1:** Characteristics of patients diagnosed with rectal neuroendocrine tumors.

Variables	SEER database		WCH database	
	*N*	Percent (%)	*N*	Percent (%)
Age				
<53	619	49.8	32	71.1
≥53	624	50.2	13	28.9
Sex				
Male	664	53.4	27	60.0
Female	579	46.6	18	40.0
Differentiation grade				
G1	1,034	83.2	31	68.9
G2	149	12.0	13	28.9
G3	60	4.8	1	2.2
T stage of primary tumor				
T1	1,115	89.8	36	80.0
T2	72	5.8	5	11.1
T3	44	3.5	1	2.2
T4	12	0.9	3	6.7
Tumor size				
<1 cm	992	79.8	22	48.9
≥1 cm	251	20.2	23	51.1
Regional lymph nodes metastasis				
Yes	71	5.7	10	22.2
No	1,172	94.3	35	77.8
Distant metastasis				
Yes	42	3.4	6	13.3
No	1,201	96.6	39	86.7
Survival months				
<25	475	38.2	–	–
≥25	768	61.8	–	–
Total	1,243	100	45	100

SEER, surveillance, epidemiology, and end results dataset; WCH, West China Hospital.

### Independent risk factors for r-LN and distant metastasis analyzed in the training cohort

**Figure 3:**
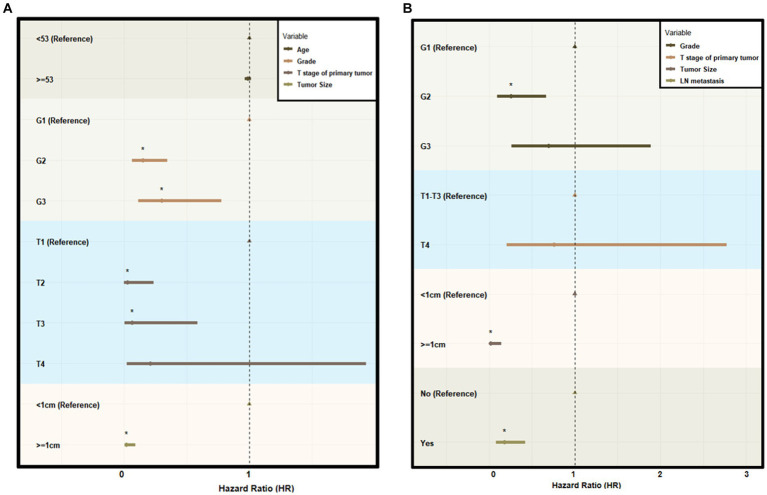
Forest plot of multivariate logistic analysis using the SEER database. **(A)** Multivariate logistic analysis of the risk of r-LN metastasis for patients diagnosed with rectal neuroendocrine tumors from the SEER database. **(B)** Multivariate logistic analysis of the risk of distant metastasis for patients diagnosed with rectal neuroendocrine tumors from the SEER database. HR indicates hazard ratio.

In the construction cohort, univariate analysis showed that sex (*p* > 0.05) was not an independent risk factor, but differentiation age, grade, tumor size and T stage may be risk factors affecting r-LN metastasis (*p* < 0.05). Following univariate analysis, a multifactorial logistic regression analysis was performed. The results revealed that age (HR = 1.027, 95% CI = 1.005–1.050), grade (HR = 0.010, 95% CI = 0.005–0.020), T stage (HR = 0.010, 95% CI = 0.000–0.012), and tumor size (HR = 0.005, 95% CI = 0.001–0.022) were independently associated with r-LN metastasis (*p* < 0.05). The results of the univariate and multifactorial analyses are shown in Table 2 and Figure 3A.

**Table 2:** Univariate and multivariate logistic analyses of the risk of r-LN metastasis in patients diagnosed with rectal neuroendocrine tumors from the SEER database.

Variables		Univariate analysis		Multivariate analysis	
		HR (95% CI)	*p*-value	HR (95% CI)	*p*-value
Age	<53	1 (Reference)	*p* < 0.05	1 (Reference)	0.43
	> = 53	1.027 (1.005–1.050)		0.989 (0.963–1.016)	
Sex	Male	1 (Reference)	0.61	–	–
	Female	1.133 (0.699–1.836)		–	–
Grade	G1	1 (Reference)	*p* < 0.05	1 (Reference)	*p* < 0.05
	G2	0.010 (0.005–0.020)		0.149 (0.064–0.346)	
	G3	0.051 (0.024–0.112)		0.300 (0.116–0.779)	
T stage of primary tumor	T1	1 (Reference)		1 (Reference)	
	T2	0.010 (0.000–0.012)	*p* < 0.05	0.027 (0.003–0.238)	*p* < 0.05
	T3	0.022 (0.003–0.184)		0.064 (0.007–0.586)	
	T4	0.159 (0.019–1.349)	0.09	0.211 (0.023–1.930)	0.17
Tumor size	<1	1 (Reference)	*p* < 0.05	1 (Reference)	*p* < 0.05
	> = 1	0.005 (0.001–0.022)		0.021 (0.005–0.093)	

HR, hazard ratio.

For the distant metastasis analysis, univariate analysis showed that age (*p* > 0.05) and sex (*p* > 0.05) were not significant risk factors in the construction cohort. The differentiation grade, tumor size, T stage and r-LN metastasis could be risk factors affecting distant metastasis. The logistic regression analysis of distant metastasis might be associated with differentiation grade (HR = 0.253, 95% CI = 0.096–0.668), tumor size (HR = 0.019, 95% CI = 0.002–0.145) and r-LN metastasis (HR = 0.179, 95% CI = 0.076–0.424), while T stage was not regarded as a meaningful factor (*p* > 0.05). The results of distant metastasis are shown in Table 3 and Figure 3B.

**Table 3:** Univariate and multivariate logistic analyses of the risk of distant metastasis in patients diagnosed with rectal neuroendocrine tumors from the SEER database.

Variables		Univariate analysis		Multivariate analysis	
		HR (95% CI)	*p*-value	HR (95% CI)	*p*-value
Age	<53	1 (Reference)	0.48	–	–
	> = 53	1.010 (0.982–1.038)		–	–
Sex	Male	1 (Reference)	0.42	–	–
	Female	1.293 (0.691–2.420)		–	–
Grade	G1	1 (Reference)	*p* < 0.05	1 (Reference)	
	G2	0.016 (0.007–0.035)		0.253 (0.096–0.668)	*p* < 0.05
	G3	0.103 (0.044–0.242)		0.697 (0.257–1.891)	0.48
T stage of primary tumor	T1 + T2 + T3	1 (Reference)	*p* < 0.05	1 (Reference)	0.67
	T4	0.030 (0.009–0.098)		0.754 (0.205–2.772)	
Tumor size	<1	1 (Reference)	*p* < 0.05	1 (Reference)	*p* < 0.05
	> = 1	0.005 (0.001–0.038)		0.019 (0.002–0.145)	
LN metastasis	No	1 (Reference)	*p* < 0.05	1 (Reference)	*p* < 0.05
	Yes	0.016 (0.008–0.033)		0.179 (0.076–0.424)	

HR, hazard ratio.

### Prognostic nomogram for r-LN metastasis

**Figure 4:**
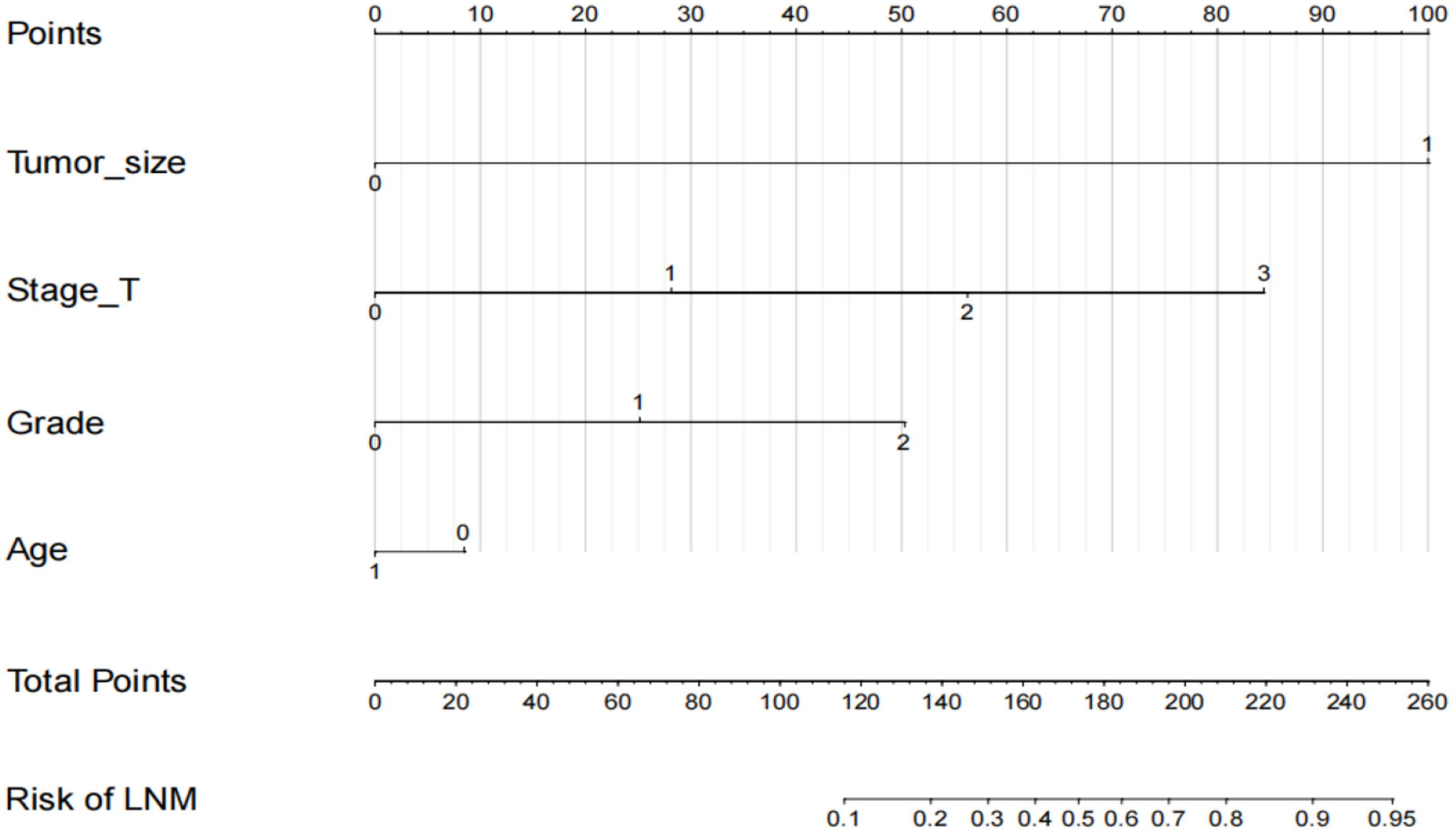
Nomogram to predict the risk of r-LN metastasis in patients with rectal neuroendocrine tumors. Points for age, tumor size, tumor grade and T stage are obtained by drawing a line upward from the corresponding values to the “Points” line. The sum of the points of these 4 factors is located on the “Total points” line.

**Figure 5:**
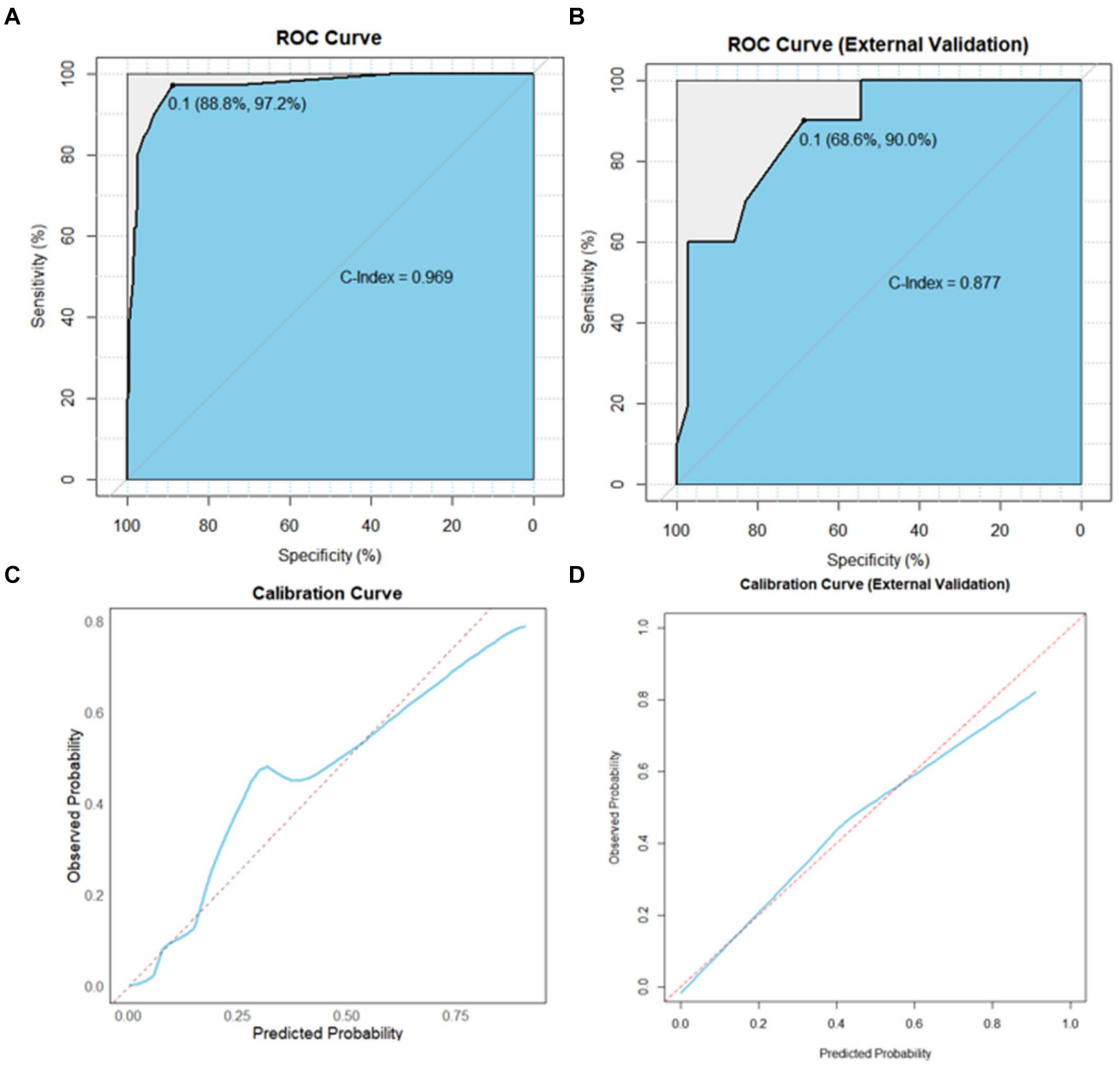
AUC value of ROC prediction for the nomogram using the training and validation sets. **(A)** Calibration plots of the nomogram for the risk of r-LN metastasis in the training set. **(B)** Calibration plots of the nomogram for the risk of r-LN metastasis in the validation set. Sky blue area under the curves of the two models to predict risk rates of r-LN metastasis. **(C)** Calibration curves in the training set. **(D)** Calibration curves in the validation set. AUC, area under the curve; ROC, receiver operating characteristic.

Based on the independent prognostic factors indicated in the multivariate logistic analysis, we developed a novel nomogram to estimate r-LN metastasis (Figure 4). Each variable was given a score according to the hazard ratio (HR). Adding up the total scores of each selected variable and locating it onto the total points scale, the probability of r-LN metastasis of an individual patient could be easily estimated. We conducted internal validation of the nomogram and obtained a C-index of 0.968 (95% CI = 0.949–0.986). By bootstrap sampling 1,000 times, the calibration curves of the nomogram were plotted and demonstrated optimal agreement between the predicted and actual survival. In the external validation using cases from the WCH database, the C-index was 0.877 (95% CI = 0.765–0.989), which showed great consistency between the nomogram prediction and the actual prognosis for the training set and validation set. The results are shown in Figures 5A,B. At the same time, the constructed calibration curves reflect the consistency between our predicted and actual values (Figures 5C,D).

## Discussion

R-NETs are generally considered tumors with a favorable prognosis, with a five-year survival rate of up to 88% in North America (15, 16). Surgery remains the primary treatment for R-NETs, with endoscopic resection often being sufficient (17). However, the choice of endoscopic surgical approach is heavily influenced by tumor size and r-LN metastasis status. Current guidelines suggest that endoscopic treatment alone is appropriate for tumors <1 cm without r-LN metastasis (18, 19). Conversely, when the tumor >1 cm, achieving R0 resection becomes challenging based on tumor size alone, requiring greater attention to r-LN involvement. Previous studies have reported that it is challenging to predict the presence of r-LN metastasis through CT scans (20). In recent years, 68Ga-DOTANOC PET-CT has been introduced in the diagnosis and treatment of neuroendocrine tumors, offering a more efficient preoperative screening for patients with positive r-LN metastasis (14). However, due to the high cost of this examination, widespread clinical implementation poses certain challenges. Therefore, our aim is to capture clinical characteristics of patients, identify those at high risk for positive r-LN metastasis, and tailor more suitable diagnostic and therapeutic strategies, thus avoiding unnecessary expenses.

Various studies have explored characteristics and risk factors for r-LN metastasis in R-NETs. Wei et al. conducted a retrospective study involving 419 patients, revealing G grade (95% CI = 2.122–32.103, *p* < 0.001), depth of tumor invasion (95% CI = 4.586–566.595, *p* < 0.001), and tumor size (95% CI = 2.798–307.298, *p* < 0.001) as independent risk factors (21). Beonghoon and his colleagues found tumor size and tumor grade to be predictive factors for LN metastasis in R-NET patients (15). A recent multicenter retrospective study in 199 patients identified tumor size >11.5 mm and vascular invasion as independent poor prognostic factors (22). Consistent with these findings, our results highlighted four factors: age, differentiation grade, T stage of primary tumor, and tumor size.

To our knowledge, this is the most extensive study on r-LN metastatic R-NETs to date, encompassing both American and Chinese cases. Despite differences between the two populations, including variations in r-LN metastasis rates (5.7% vs. 22.2%), the predictive model we developed performed well in the validation cohort. Our study identified four independent prognostic factors and introduced a novel nomogram. Current guidelines for R-NETs recommend a formal oncologic low anterior resection with total mesorectal excision based on patients’ clinical and genetic characteristics (9, 23). Management should be guided by predictors of nodal involvement (24, 25). Our study aims to enhance the recognition of r-LN metastasis, aiding clinicians in estimating optimal treatment. Additionally, this is the first study constructing an r-LN metastasis nomogram based on both American and Chinese R-NET patients.

However, some limitations exist. Firstly, due to its retrospective nature, our study is susceptible to selection bias. Secondly, unavailable data in the SEER database might lead to the oversight of crucial factors such as depth of tumor invasion, vascular invasion, ki-67, etc. Thirdly, owing to the low incidence of R-NETs, the validation cohort’s sample size was small. Despite the limitations of this study, we advocate for larger prospective studies to further validate our findings. Additionally, incorporating more clinical features, such as genetic markers and lifestyle factors, into the model assessment is essential to enhance the nomogram’s adaptability to real-time clinical practice.

## Conclusion

We identified four independent LN metastasis risk factors in a large cohort of R-NET patients, including age, differentiation grade, T stage of primary tumor and tumor size. In addition, a novel nomogram was developed based on these variables. Internal validation of the nomogram exhibited satisfactory performance. Further research is needed to verify the practicality of this model.

## Data Availability

The raw data supporting the conclusions of this article will be made available by the authors, without undue reservation.

## Footnotes

^1^ http://www.r-project.org/
